# Analysis of Cardiac Arrhythmias Based on ResNet-ICBAM-2DCNN Dual-Channel Feature Fusion

**DOI:** 10.3390/s25030661

**Published:** 2025-01-23

**Authors:** Chuanjiang Wang, Junhao Ma, Guohui Wei, Xiujuan Sun

**Affiliations:** 1 College of Electrical Engineering and Automation, Shandong University of Science and Technology, Qingdao 266590, China; cxjwang@sdust.edu.cn (C.W.); 202382080046@sdust.edu.cn (J.M.); 2 Zhuhai Inpower Electric Co., Ltd., Zhuhai 519000, China; weiguohui97@gmail.com

**Keywords:** arrhythmia, abnormal ECG signal detection, deep learning, feature fusion, attention mechanism

## Abstract

Cardiovascular disease (CVD) poses a significant challenge to global health, with cardiac arrhythmia representing one of its most prevalent manifestations. The timely and precise classification of arrhythmias is critical for the effective management of CVD. This study introduces an innovative approach to enhancing arrhythmia classification accuracy through advanced Electrocardiogram (ECG) signal processing. We propose a dual-channel feature fusion strategy designed to enhance the precision and objectivity of ECG analysis. Initially, we apply an Improved Complete Ensemble Empirical Mode Decomposition with Adaptive Noise (ICEEMDAN) and enhanced wavelet thresholding for robust noise reduction. Subsequently, in the primary channel, region of interest features are emphasized using a ResNet-ICBAM network model for feature extraction. In parallel, the secondary channel transforms 1D ECG signals into Gram angular difference field (GADF), Markov transition field (MTF), and recurrence plot (RP) representations, which are then subjected to two-dimensional convolutional neural network (2D-CNN) feature extraction. Post-extraction, the features from both channels are fused and classified. When evaluated on the MIT-BIH database, our method achieves a classification accuracy of 97.80%. Compared to other methods, our approach of two-channel feature fusion has a significant improvement in overall performance by adding a 2D convolutional network. This methodology represents a substantial advancement in ECG signal processing, offering significant potential for clinical applications and improving patient care efficiency and accuracy.

## 1. Introduction

Cardiovascular disease, as a noncommunicable disease, has always occupied the first place in mortality due to its insidious nature and long-term nature. Indeed, substantial health care with regards to the burden of CVD is not denied. In 2017, there were around 483 million people suffering from this disease all over the world, representing 6.4% of the total global population. In the same year, 17.7 million deaths accounted for CVDs, which took up approximately 32% of all the deaths globally [1]. According to the official statistics of China in 2020, the number of patients suffering from cardiovascular diseases in China is about 330 million. Meanwhile, in 2020, 45.86% of total deaths in urban areas and 39.9% of the total deaths in rural areas are related to cardiovascular diseases. furthermore, the incidence trend of CVD has been becoming younger and younger [2].

A study estimated the indirect costs of heart disease among Australian working adults ages 45 to 64 years. The model estimated that Australia lost AUD 785 million (approximately USD 755 million) in GDP in 2015 due to lower incomes, higher welfare spending, and lower income tax revenues. It is expected that this loss in GDP could further increase and rise to AUD 1125 million (approximately USD 1082 million) by the year 2030 [3]. Thus, CVD has a deep impact both on the well-being and health of a person and the economy in general.

At present, the most common method for determining arrhythmia in clinical practice involves examining the Electrocardiogram (ECG) by a physician or specialist manually [4]. The results of such examinations are often largely influenced by the doctor’s or expert’s subjectivity. In the meantime, deep learning has led to significant growth in the medical field. Guo et al. created an evolutionary convolutional neural network that automatically finds the best architecture for EEG emotion recognition. It achieved 96.47% accuracy on the DEAP dataset and competitive results on the Dreamer dataset [5]. David and his colleagues proposed a method for detecting lung diseases called DIWGAN-ROA-LDD-CXRI. This method is based on a dual interactive Wasserstein generative adversarial network (DIWGAN) and is optimized using a remora optimization algorithm. It is designed to classify chest X-ray images into three categories: COVID-19, normal, and pneumonia cases. This method reduces the calculation time and improves the classification accuracy [6]. Chen et al. proposed a deep convolution network (deep DSP) combined with a double spatial pyramid structure for tongue image segmentation. The method embedded the spatial pyramid module in the encoder–decoder architecture and applied the novel double spatial pyramid architecture to learn the multi-coding state features. The experimental results show that deep DSP is superior to the existing advanced methods in accuracy, recall rate, F1 value, and specificity [7]. Therefore, it is of great significance to develop an automatic rhythm recognition and classification system that combines existing computer technology and artificial intelligence technology [8]. It is an important tool for monitoring heart health, which is efficient, accurate, and fast. Thus, automatic rhythm recognition and classification systems have become an indispensable trend in the application of ECG examinations.

A great deal of work and research is being conducted to create and develop a stable, reliable, and accurate system for detecting and analyzing ECG signals [9,10]. Preprocessing of ECG signals is crucial as effective denoising directly impacts system performance. In recent years, researchers at home and abroad have conducted a lot of studies in the field of ECG signal denoising, which significantly improved the denoising quality [11,12,13]. However, there may be some residual EEMD noise problems and spurious modes in the initial decomposition stage, so this study proposes an ECG signal denoising method based on the combination of ICICEMDAN and improved wavelet thresholding, which can effectively suppress the above problems.

In deep learning-based arrhythmia analysis, feature extraction and classification of ECG signals complement each other, with many methods emerging for reference in recent years [14,15]. Currently, ECG signal processing methods share some common core features, including the application of mathematical transforms, feature extraction, and compression techniques, the possibility of multiscale analysis, and signal enhancement means. These features reflect the current dominant trends and technological tools in the field of ECG signal processing [16,17,18]. In the field of ECG signal classification and identification, machine learning algorithms, commonly used as classifiers, include logistic regression, random forests, support vector machines, clustering, and artificial neural networks [19]. Taiyoung Li employed a strategy that combined wavelet decomposition with random forests, achieving an impressive accuracy of 94.61% in the classification of arrhythmias. This approach leveraged the strengths of wavelet decomposition for processing ECG signals along with the robust learning capabilities of random forests. This method demonstrated a significant improvement in performance for arrhythmia classification compared to previous studies. The findings of this research offer an effective approach in the field of ECG signal analysis and serve as a valuable reference for future studies on similar topics [20]. Sahoo et al. used an artificial neural network (ANN) combined with a modified genetic algorithm (MGA) to optimize the weighting parameters and achieved an arrhythmia classification accuracy of 90.00% [21]. Xu used features based on RR intervals and discrete wavelet decomposition and used an extreme learning machine as a classifier due to its advantages in handling high dimensional data and generalization performance [22]. Gaetano designed a supervised neural network model using radial basis functions to process raw signals and extract feature vectors, which is suitable for binary classification of normal and ischemic ECG signals but has not been widely validated for multi-classification problems [23]. Essa combines convolutional neural networks with long- and short-term memory networks to extract features automatically, which achieves 95.81% accuracy, but there are problems of function redundancy and increased time cost [24].

To address the shortcomings of previous studies, this paper adds the improved hybrid attention ICBAM mechanism module within each residual module, based on the existing ResNet. The features from the one-dimensional ResNet-ICBAM network structure for the ECG signal are extracted. Features from two-dimensional convolutional neural networks for GADF, MTF, and RP images, transformed into RGB format from the ECG signal, are also extracted. The extracted features are then fused and fed into a classifier for classification detection. This process results in excellent performance and significantly enhances the recognition effect.

The remainder of the paper is organized as follows. Section 2 details the research methodology, including preprocessing techniques, network architecture enhancements, and dual-channel feature fusion. Section 3 outlines the experimental setup and results, followed by conclusions and future research directions in Section 4.

## 2. Methodology

The workflow first extracts and preprocesses ECG signals from the MIT-BIH arrhythmia database by using the ICEEMDAN algorithm combined with enhanced wavelet thresholding for noise reduction. Subsequently, the signal is segmented into 5 s segments and classified into five categories according to the AAMI standard. To solve the category imbalance problem, the SMOTE algorithm is applied to oversample a few categories. Subsequently, one-dimensional ECG signals are converted to two-dimensional representations (GADF, MTF, RP) to enhance feature extraction. A two-channel feature fusion model, ResNet-ICBAM-2DCNN, is constructed. The first channel processed the 1D ECG signals using the ResNet-ICBAM network, and the second channel extracted features from the 2D images via 2D-CNN. Finally, the fused features are classified using the Softmax classifier.

### 2.1. ECG Signal Preprocessing

#### 2.1.1. ECG Signal Denoising

Noise in ECG signals, often due to acquisition and storage issues, degrades signal quality. Analyzing such noisy signals can reduce accuracy and affect model performance, underscoring the importance of denoising in ECG abnormality detection and classification. This section introduces a denoising method combining improved wavelet thresholding with ICEEMDAN.

The ICEEMDAN algorithm decomposes the signal in the following steps:

Step 1: The decomposition sequence x(t) is formed by adding white noise to the signal $x_{i}(t)$ to be decomposed, and then the EMD algorithm is applied to process it to obtain the 1st-order residual component $r_{1}(t)$:

(1) $$x_{i}=x(t)+\alpha_{0}E_{1}(\delta_{i})$$

(2) $$E_{1}(x_{i}(t))=x(t)-M(x_{i}(t))$$

(3) $$r_{1}(t)=AVERAGE[M(x_{i}(t))]$$

The signal obtained after the EMD algorithm is composed of a series of components, denoted by $E_{k}(\cdot )$. The local mean processing of the signal is represented by M(·). The noise standard deviation is indicated by $\alpha_{k}$, and the ith white noise added is denoted by $\delta_{i}$.

Step 2: The first modal component $I_{IMF1}(t)$ for k = 1 is calculated:

(4) $$I_{IMF1}(t)=x(t)-r_{1}(t)$$

Step 3: The residual component $r_{2}(t)$ for k = 2 and the 2nd modal component $I_{IMF2}(t)$ is calculated:

(5) $$I_{IMF2}(t)=r_{1}(t)-r_{2}(t)$$

(6) $$r_{2}(t)=AVERAGE[M(r_{1}(t)+\alpha_{1}E_{2}(\delta_{i}))]$$

Step 4: The kth order residual component $r_{k}(t)$ and the kth modal component $I_{IMFk}(t)$ are calculated:

(7) $$I_{IMFk}(t)=r_{k-1}(t)-r_{k}(t)$$

(8) $$r_{k}(t)=AVERAGE[M(r_{k-1}(t)+\alpha_{k-1}E_{k}(\delta_{i}))]$$

Step 5: Steps one through four are repeated to obtain all the residual and modal components.

In the context of wavelet thresholding algorithms, hard thresholding introduces discontinuities, complicating practical implementation, while soft thresholding causes deviations in wavelet coefficients, leading to signal loss [25]. To solve this problem, a new threshold function is adopted for processing, and its formula is shown below:

(9) $$f(x)=\left\{\begin{array}{l} sign(x)\left[\left|x\right|-\frac{2T}{\exp[\frac{\left|x\right|-T}{T}]+1}\right],\;\left|x\right|\geq T\\ 0,\;\left|x\right|<T \end{array}\right.$$

When |*x*| is smaller than the threshold T, the wavelet domain will be all set to 0, When $\left|x\right|\to T$, *f(x)* will be infinitely close to $sign(x)\left[\left|x\right|-T\right]$, at which time the improved wavelet threshold function can be approximated as a soft threshold function, and when |*x*| is larger than the threshold *T*, *f(x)* tends to be equal to *x*, at which time the improved wavelet threshold function is approximated as a hard threshold function.

**Figure 1:**
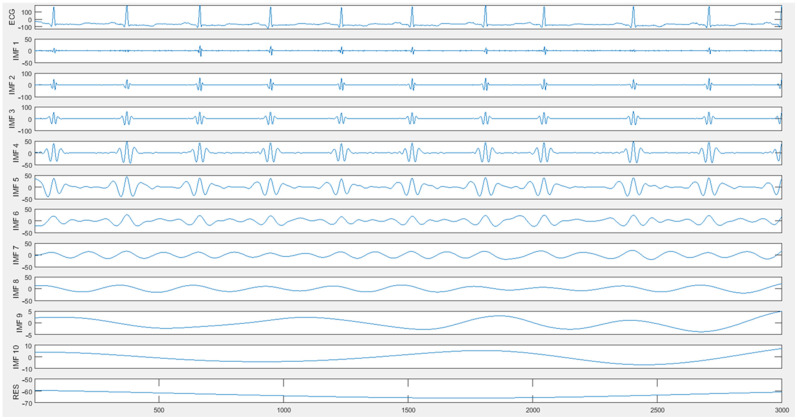
ICEEMDAN decomposition of the raw ECG signal.

The first 3000 sampling points of record 100 of the MIT-BIH database were selected, and after ICEEMDAN decomposition, the signal was decomposed into 10 IMF modal components and 1 RES residual component, as shown in Figure 1.

**Figure 2:**
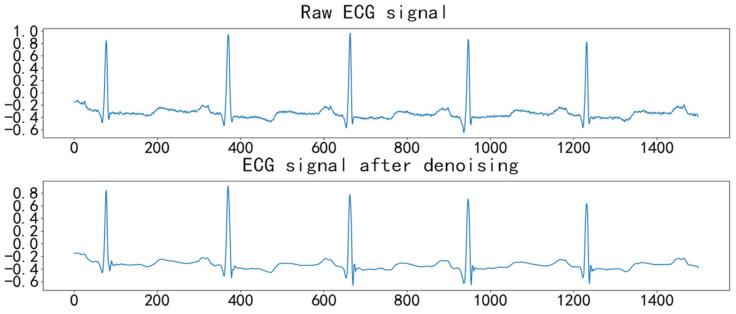
Comparison of the denoising effect of ECG signals.

Using the ICEEMDAN method, we decomposed the ECG signals into high- and low-frequency components. High-frequency components were processed with a modified wavelet thresholding method using the sym8 wavelet over five layers. Low-frequency components, which included baseline drift noise below 1.5 Hz, were cleaned by setting a threshold of 1.5 Hz. The signal was then reconstructed from the processed components. By denoising the ECG signal with a sampling length of 1500, the comparison of its effect before and after denoising is shown in Figure 2.

The results show that the proposed denoising method performs well in the performance evaluation. The mean values of the output signal-to-noise ratio (SNR) range from 26.36 dB to 28.23 dB, the mean value of the root-mean-square error (RMSE) is lower than 0.034, and the waveform similarity coefficient (NCC) is higher than 0.998. This denoising method has a significant superiority in effectively removing the noise while better preserving the original features of the ECG signal.

#### 2.1.2. Slicing and Processing of ECG Signals

In this study, the following rules were used to determine the type of ECG signal slices:

(1) A slice is normal if all heartbeats in the slice are of the normal type.
(2) If a slice has both a normal heartbeat and an abnormal heartbeat, the type of the slice is the type of this abnormality.
(3) If more than one type of anomaly heartbeat exists in a slice at the same time, the type of anomaly with the highest number of them will determine the type of the slice.
(4) If there are multiple abnormal heartbeats in a slice at the same time and their number is the same, the type of abnormality that occurs first is used as the type of that slice.

This method solves the problem of needing to rely on the QRS wave detection algorithm to locate the R-wave position and solves the problem of unbalanced data samples by overlapping slices. By overlapping between neighboring slices, the number of samples can be increased, and the quality of samples can be improved [26].

#### 2.1.3. SMOTE Algorithm Data Enhancement

Solving the problem of sample data imbalance is crucial to improving the recognition accuracy of various types of ECG signals.

**Figure 3:**
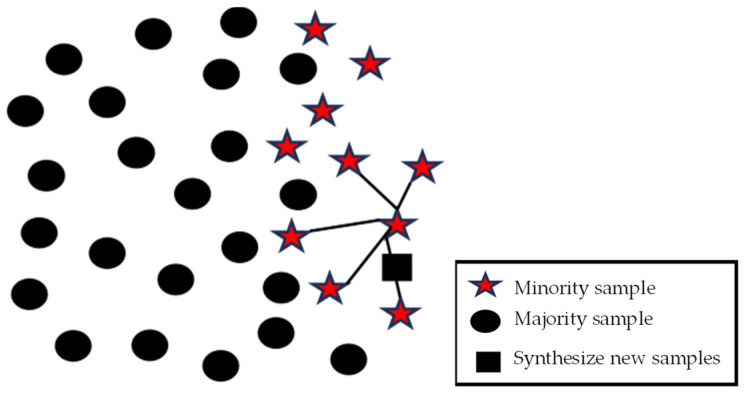
**The** SMOTE oversampling method to generate new sample data.

SMOTE, the synthetic minority oversampling technique, improves random oversampling. The core idea of the SMOTE algorithm is to use the sample data of the minority class as a template and generate new sample data randomly without making changes to the sample data of the minority class, thus reducing the impact of data imbalance in the dataset to a certain extent [27]. The schematic diagram of the SMOTE oversampling method for generating new sample data is shown in Figure 3.

When using the SMOTE oversampling method for data enhancement, 20% of the dataset was first classified as the test set to ensure the accuracy of the test results, data enhancement was performed on the rest of the data, and no processing was performed on the normal type of ECG signal. Table 1 shows the before and after comparison of the number of samples for data enhancement using the SMOTE method.

After data enhancement using the SMOTE oversampling method, the number of data types other than the normal type of ECG signal is greatly increased, which solves the problem of imbalance for each kind of data between the datasets to a certain extent and makes the ratio of the number of each type in the ECG signal data 1:1:1:1:1.

### 2.2. Graphical Processing of ECG Signals

In ECG anomaly recognition, Ahmad et al. [28] created a new image fusion model for classifying ECG beats. The model utilized GAF, RP, and MTF to convert the one-dimensional heartbeat signals into three-channel 2D images to enhance feature representation and achieve efficient and accurate classification by deep learning techniques. In addition, Cai et al. [29] adopted a similar strategy in their study where they used GAF, RP, and tiling to create three-channel embeddings for ECG signal classification tasks. These methods effectively improved the quality of the feature representation and, hence, the classification performance. It is shown that the use of GAF, RP, and MTF can effectively enhance the feature representation of ECG signals and, thus, improve the recognition of ECG abnormalities. Therefore, in this paper, the ECG signals are transformed into a Gram angle field, Markov migration field, and recurrence map, respectively, to better extract the features of ECG signals.

**Figure 4:**
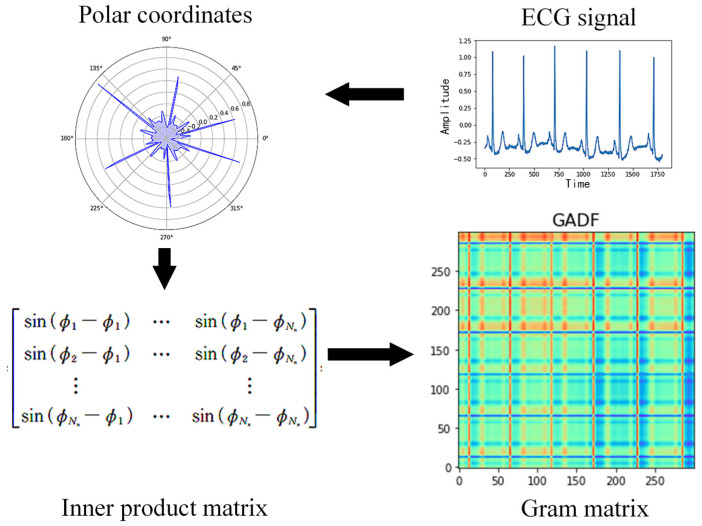
A schematic diagram of Gramm’s angle field generation.

GAF is a technique for converting a one-dimensional time series into two-dimensional data, preserving the signal information and time dependence intact. The idea is to convert the signal from a right-angle coordinate system to a polar coordinate system, and then use the inner product matrix to compute the feature matrix to generate a two-dimensional image. The process of its transformation is shown in Figure 4.

**Figure 5:**
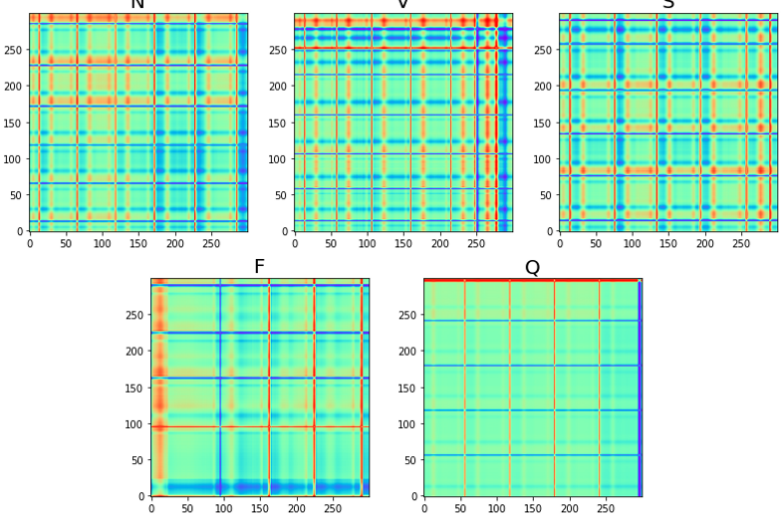
GADF for each type of ECG signal.

As the ECG signal needs to focus on local variations, the Gram angular difference field is used in this study. Its symmetry is reflected in the fact that in the Gram angular difference field, all elements except for the diagonal elements are opposite each other about the diagonal. The diagonal data are 0, and the angles corresponding to 1, 0, and −1 are 90°, 0°, and −90°, indicating the peaks and troughs. The results of the five types of ECG signals transformed by the Gram angle difference field are shown in Figure 5.

**Figure 6:**
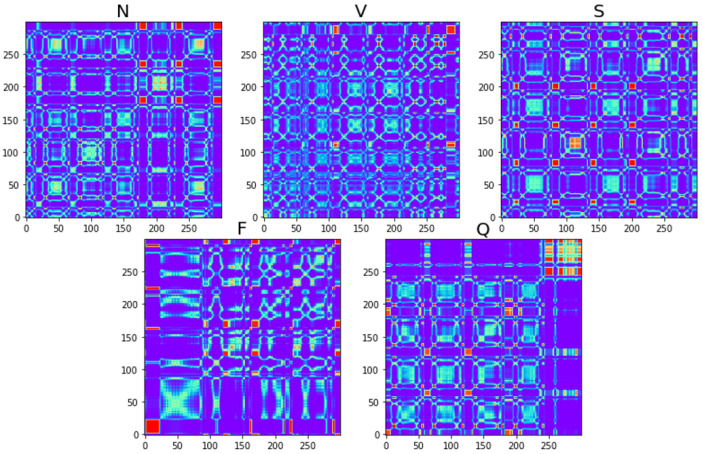
MTF for each type of ECG signal.

In this study, the idea of using the graphical representation of the Markov variation field of the ECG signals can be briefly described as follows: firstly, the ECG signals are normalized; then the normalized ECG signals are used to generate the Markov migration matrix; and then the Markov migration matrix is used to generate the Markov variation matrix, which is then used for the final visualization of the pictures. The transformed Markov shift fields for the five types of ECG signals are shown in Figure 6.

RP is a method of visualizing and analyzing a time series, transforming the signals of the time series into graphs and visualizing them to make it easier to analyze the patterns and structure of the signals in the time series. The formula for the vector is:

(10) $$x_{i}=[{\tilde{x}}_{i},{\tilde{x}}_{i+\tau },\cdots ,{\tilde{x}}_{i+(m-1)\tau }],\;i=1,2,\cdots ,n-(m-1)\tau$$

**Figure 7:**
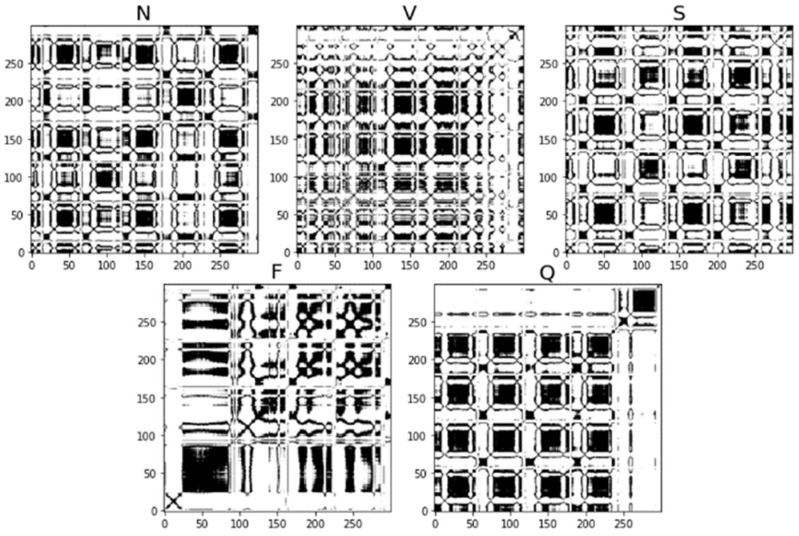
RP for each type of ECG signal.

Let H(·) be the unit step function and ε be the threshold, then the expression of the recurrence map is:

(11) $$R_{\left(i,j\right)}=H(\varepsilon -\left\|x_{i}-x_{j}\right\|),\;i=1,2,\cdots ,n-(m-1)\tau \;\;j=1,2,\cdots ,n-(m-1)\tau$$

where the delay parameter is τ, m is the embedding dimension, and the given time series is normalized to $x=[x_{1},x_{2},\cdots ,x_{i},\cdots ,x_{n}]$. In this study, the threshold is selected as ε=30, and the recurrence diagram transformed by the five types of ECG signals is shown in Figure 7.

### 2.3. Deep Learning Models

ResNet solves the gradient degradation problem in deep networks through the residual structure, while ICBAM enhances the feature representation and classification accuracy through the channel attention and spatial attention mechanisms, optimizes the resource utilization, and improves the model robustness. The ResNet-ICBAM model combines the strengths of the two and dynamically adjusts the feature weights by embedding the ICBAM in the residual module of ResNet, thus improving the performance of arrhythmia detection and classification.

#### 2.3.1. ResNet Network Model

The ResNet network model with residual structure shows great advantages in solving the degradation problem of deep learning network models due to its internal batch normalization operation, thus successfully solving the problem that the gradient update either grows exponentially or decays exponentially during the training process. According to the number of layers of different ResNet network models, ResNet can be classified as ResNet-18, ResNet-34, ResNet-50, ResNet-101, etc. The ResNet network architecture is shown in Table 2.

There are two types of residual modules: shallow residual module structures and deep residual module structures. The residual modules of ResNet-18 and ResNet-34 are shallow residual structures, and the residual module structures of ResNet-50 and ResNet-101 are deep residual structures [30]. Since the ECG signal contains fewer digits per slice and the net18 network model is used in this study, each residual module is halved to reduce the amount of computation for lightweight operations.

#### 2.3.2. ICBAM Model

The integration of ICBAM into the model has been shown to enhance the feature representation and classification accuracy of the model. By integrating the channel attention and spatial attention mechanisms, ICBAM enables the network to dynamically adjust the feature weights and focus on important information, thereby improving the model’s ability to capture subtle differences in ECG signals. Furthermore, ICBAM optimizes resource utilization, reduces computation, and enhances the robustness and adaptability of the model, particularly in the presence of noise interference or variations in signal characteristics.

**Figure 8:**
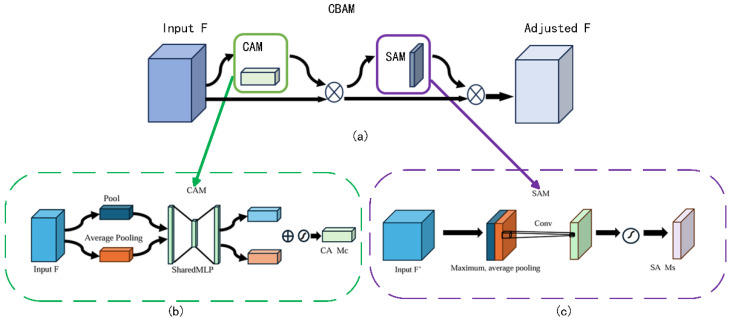
Components of the CBAM network structure. (**a**) CBAM network structure diagram; (**b**) channel attention module; (**c**) space attention module.

The convolutional block attention module (CBAM) consists of two parts: the channel attention module (CAM) and the space attention module (SAM). Its structure is shown in Figure 8a.

The CAM and the SAM constitute the components of the CBAM. Initially, global pooling and average pooling operations are performed on the input feature maps, respectively. Subsequently, the pooling results are fed into the multilayer shared perceptual machine (MLP). Following this, arithmetic operations are performed, and the obtained results are summed. Finally, the results are mapped by the sigmoid function to obtain the results, as illustrated in Figure 8b.

The output of the channel attention module CAM is then taken as its input, and global pooling and average pooling operations are performed on the input. The obtained results are stitched according to the channels, and the results are obtained by activation function processing of the convolution results. The CAM’s output is then utilized as the input for global and average pooling operations. The spliced results are then processed through convolution operations, followed by activation function processing of the convolution results. The results are obtained through this sequence of operations [31]. As demonstrated in Figure 8c, the resultant output can be obtained by processing the convolution result through the activation function.

**Figure 9:**
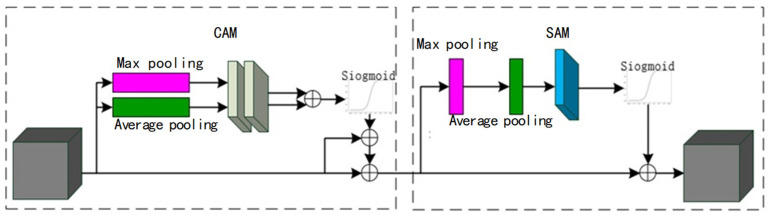
ICBAM attention mechanism structure diagram.

The problem of spatial information loss is solved by adding the results of the channel attention module operation to the spatial attention module and reusing the global features of the backbone network output. This improved hybrid attention module mechanism is named ICBAM and its structure is shown in Figure 9.

ICBAM enhances the feature map through two modules, channel attention and spatial attention. The channel attention module first averages and maximally pools the feature maps, then processes them through fully connected layers and Sigmoid activation functions, and finally sums them with the original feature maps to obtain the enhanced feature maps. The spatial attention module, on the other hand, averages and maximally pools the feature maps before normalizing them by convolution and Sigmoid activation function to highlight important spatial regions. In the channel attention module, the process can be represented by the following equation:

(12) $$F_{avg}^{c}=\frac{1}{W*H}\sum_{i=1}^{W}\sum_{j=1}^{H}F(i,j)$$

(13) $$F_{\max}^{c}=\max(F(i,j);i\in W,j\in H)$$

(14) Wc=Sigmoid(FC2(ReLU(FC1(Favgc+Fmaxc))))

(15) $$F_{1}=W_{out}^{c}=F*W^{c}+F$$

In the spatial attention module, the process can be expressed by the following equation:

(16) $$F_{2}=W_{out}^{s}=F_{1}*Conv(mean(\max(F_{1})))$$

where *W* and *H* represent the width and height of the input feature map; *FC*_1_, and *FC*_2_ represent the two connectivity layers, respectively; the output of the channel attention module is *F*_1_; and the output of the spatial attention module is *F*_2_.

#### 2.3.3. ResNet-ICBAM Network Model

**Figure 10:**
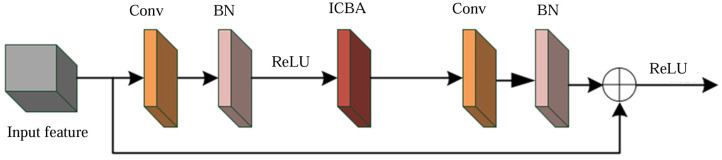
A residual module with ICBAM.

In this paper, we propose the ResNet-ICBAM network model, which combines the improved convolutional block attention module ICBAM with the residual network ResNet for arrhythmia detection and classification. Given that the ECG signal is one-dimensional, a one-dimensional neural network approach is used. In the residual module of ResNet, ICBAM is added before the second convolutional layer to enhance the model performance. After incorporating the ICBAM, the structure of the residual module is modified. This modified structure is shown in Figure 10.

**Figure 11:**
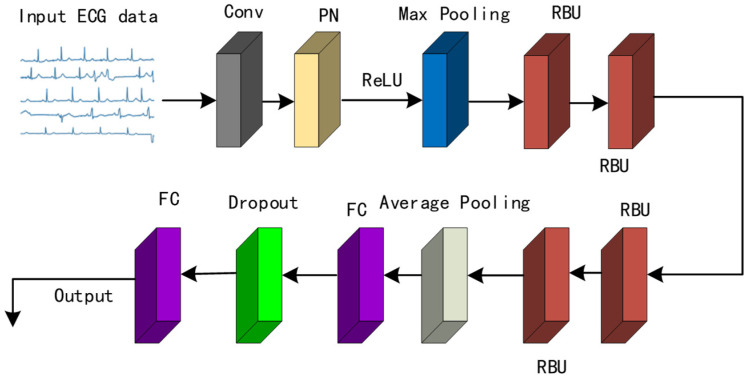
The structure of the ResNet-ICBAM network model.

ICBAM dynamically adjusts the weights of channel and spatial features, allowing the network to focus more on the important features and enhance its feature representation. Adding ICBAM to the residual module helps the network pay more attention to the key features of the ECG signals and enhances its representation capability. In addition, ICBAM makes the feature representation more discriminative, improves the model generalization ability, reduces overfitting, and makes the model perform better on new data. Figure 11. shows the network structure of ResNet-ICBAM.

### 2.4. Dual-Channel Feature Fusion Network Model Architecture Design

Recent studies have also demonstrated the effectiveness of combining deep learning with other optimization techniques for feature fusion. Singh et al. proposed a hybrid optimization method combining deep learning and weighted nonlinear regression for pan-sharpening, achieving significant improvements in image resolution [32]. Their approach fuses deep max-out network (DMN) results with a weighted nonlinear regression model. This highlights the potential of combining deep learning with other optimization techniques to improve feature representation and model performance, which aligns with the dual-channel feature fusion approach proposed in this study.

**Figure 12:**
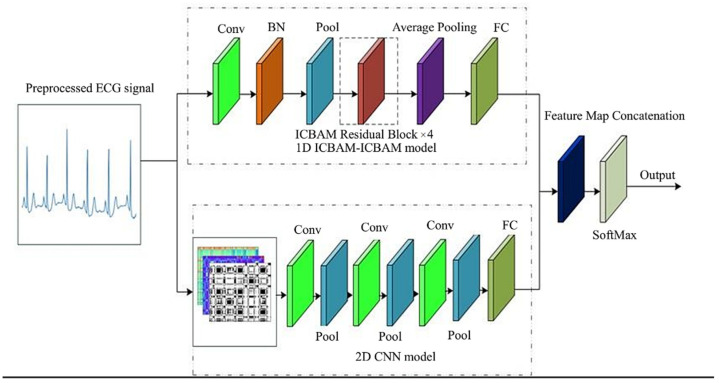
A schematic diagram of the structure of the ResNet-ICBAM-2DCNN model.

The general structure schematic of the dual-channel feature fusion ResNet-ICBAM-2DCNN built in this paper is shown in Figure 12.

The first channel uses a one-dimensional ResNet-ICBAM network for feature extraction of the 5 s ECG signal, using the ICBAM mechanism to focus on learning the useful features in the region of interest and mining the deep features of the ECG signal. The second channel uses a 2D convolutional neural network to process the Gram angular difference field, Markov variation field, and recurrence map of the ECG signal, which is transformed into an RGB image for training to extract the features of the ECG signal. Finally, the features of the two channels are spliced and fused, and the prediction discrimination is performed using a fully connected layer and a Softmax classifier. The specific steps are as follows:

(1) First channel: the ECG signal is processed for 5 s (360 Hz, 1800 data points) using a one-dimensional ResNet-ICBAM network, spatial morphology features are extracted by a one-dimensional convolutional neural network, and the focus is on the important regions using the ICBAM attention mechanism.
(2) Second channel: the ECG signal is transformed into a Gram angular difference field, Markov variation field, and recurrence map, compressed to 100 × 100 size, combined into RGB image (100 × 100 × 3), and fed into a 2D convolutional neural network to extract features.
(3) Fusion and classification: the features of the two channels are spliced and fused and sent to the Softmax classifier for classification prediction through the fully connected layer.

The parameter design of the network layer of the ResNet-ICBAM-2DCNN network model is shown in Table 3.

Let the output of the first channel of the two-channel feature fusion ResNet-ICBAM-2DCNN network model be $X^{1st}=(x_{1}^{1st},x_{2}^{1st},\cdots ,x_{256}^{1st})$, and the output of the second channel be $X^{2\mathrm{nd}}=(x_{1}^{2nd},x_{2}^{2nd},\cdots ,x_{144}^{2nd})$, which is spliced by the splicing function to achieve the purpose of feature fusion, and let *X* be the fused feature, which is calculated by

(17) $$\begin{array}{l} X={\mathrm{concat}(X}^{1st}{,X}^{2nd})\\ \;\;\;=\;(x_{1}^{1st},x_{2}^{1st},\cdots ,x_{256}^{1st},x_{1}^{2nd},x_{2}^{2nd},\cdots ,x_{144}^{2nd}) \end{array}$$

Inside the *X*, there are 400 data points, and finally, the fused features are transmitted to the Softmax classifier through the fully connected layer, which can complete the classification and detection. By using this method, different features can complement each other’s strengths, addressing the limitations of relying on a single feature. This approach enhances the performance of the network model in detecting and classifying ECG signals, significantly improving both accuracy and generalization.

## 3. Experimental Process and Result Analysis

### 3.1. Training Process and Hyperparameters

#### 3.1.1. Performance Evaluation Indicators

To assess the classification model’s ability to predict new data, i.e., its generalization ability, this paper employs a series of performance metrics for analysis and comparison, including the confusion matrix (CM), accuracy (Acc), precision (PPV), sensitivity (Sen), specificity (Spec), F1 score (F1), and overall accuracy (OA). The following is an overview of these metrics:

The confusion matrix is an intuitive tool for comparing the true categories of the data with the model predictions to assess how correctly and incorrectly each category is categorized. For binary classification problems, it is usually represented by a 2 × 2 matrix, as shown in Table 4.

TP and TN represent the true positive and true negative categories, respectively. Conversely, FP and FN refer to the false positive and false negative categories. In this context, “T” indicates that the true label aligns with the prediction, while “F” shows that it does not. Additionally, “P” denotes a positive prediction, and “N” signifies a negative prediction.

Accuracy (Acc) measures the proportion of correct predictions out of all predictions. The mathematical expression is:

(18) $$Acc=\frac{TP_{n}+TN_{n}}{TP_{n}+TN_{n}+FP_{n}+FN_{n}}\times 100\%$$

Precision (PPV) measures the proportion of all samples predicted to be in the positive category that are in the positive category. The mathematical expression is:

(19) $$PPV=\frac{TP_{n}}{TP_{n}+FP_{n}}\times 100\%$$

Sensitivity (Sen) measures the proportion of all true positive class samples that are correctly identified. The mathematical expression is:

(20) $$Sen=\frac{TP_{n}}{TP_{n}+FN_{n}}\times 100\%$$

Specificity (Spec) measures the proportion of all true negative class samples that are correctly predicted to be negative. The mathematical expression is:

(21) $$Spec=\frac{TN_{n}}{TN_{n}+FP_{n}}\times 100\%$$

F1 Score (F1) combines accuracy and sensitivity to provide a single score that balances the two. The mathematical expression is:

(22) $$F1=\frac{2\times PPV_{n}\times Sen_{n}}{PPV_{n}+Sen_{n}}\times 100\%$$

Overall accuracy (OA) reflects the overall prediction accuracy of the model over the entire dataset. The mathematical expression is:

(23) $$OA=\frac{TP+TN}{TP+TN+FP+FN}\times 100\%$$

#### 3.1.2. MIT-BIH Database

The MIT-BIH database, created by a collaboration between MIT and Beth Israel Medical Center, contains 48 recordings of dual-channel ECG signals from 47 patients (25 males and 22 females), each of more than 30 minutes’ duration, at a sampling frequency of 360 Hz. At least two physicians have annotated the ECG signal in the database and over 110,000 annotations are included. The data file format is Format 212 and contains [.hea] header files, [.dat] data files, and [.art] annotation files, which store the sampling frequency, lead information, and expert-annotated ECG signal types and R-wave positions, respectively [33].

**Figure 13:**
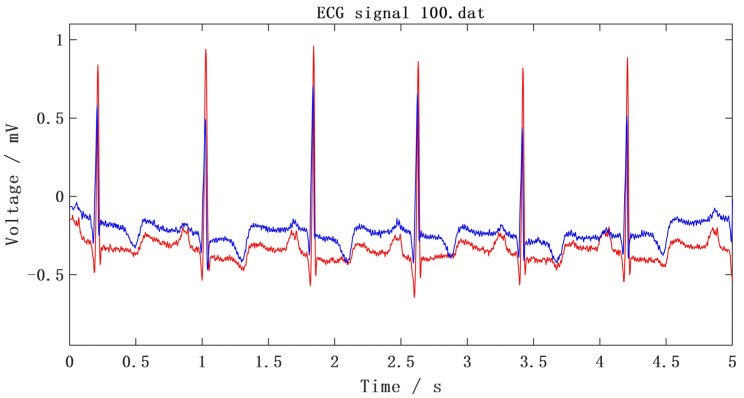
ECG waveform of No. 100 record. where the red lines indicate MLII leads and the blue lines V5 leads.

All ECG signals used in this experiment are from the MIT-BIH arrhythmia database. The MIT-BIH database has 48 double-lead ECG records with a length of 30 min and a sampling frequency of 360 Hz [34]. The data are from 47 testers. The ECG waveform diagram of the first 2000 samples recorded in database No. 100 is shown in Figure 13.

Following the denoising and slicing of the ECG signal, a total of 34,945 ECG signal samples were obtained. Of these, 20% (6989 samples) were discarded, leaving 27,956 samples. The enhancement of the dataset resulted in the acquisition of 47,015 additional ECG samples. The validation set, which comprises 30% of the samples obtained after data enhancement, and the training set, which comprises the remaining 70% of the data, are used for this study. The number of validation sets is 14,105, and the number of training sets is 32910. It should be noted that each ECG signal sample contains 1800 data points.

In this study, the AAMI standard was adopted for the classification of ECG signal beats, which categorizes the ECG signals into five major types: normal or bundle branch block beats (N), supraventricular abnormal beats (S), ventricular abnormal beats (V), fusion beats (F), and unclassifiable beats (Q) [35]. The AAMI standard and the beat types annotated in the MIT-BIH database represent two distinct classification methods in clinical practice. Therefore, to conduct arrhythmia detection and classification research using the AAMI standard on the MIT-BIH database, it is necessary to convert the beat types annotated in the MIT-BIH database into the categories specified by the AAMI standard.

#### 3.1.3. Training Process

This experiment was conducted on a server equipped with a specific hardware and software environment. The configuration of the experimental equipment is as follows: the operating system is Ubuntu 18.04.5 LTS, the processor is Intel(R) Xeon(R) Gold 6226R CPU @ 2.90GHz, and the graphics card is Tesla V100S-PCIE-32GB. The programming language is Python 3.8, and the deep learning framework is based on Pytorch 1.7.0+cu110. 1.7.0+cu110. This setup guarantees the experiment runs efficiently and produces reliable results.

**Figure 14:**
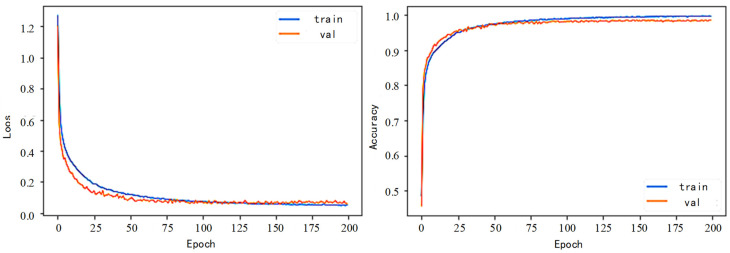
ResNet-ICBAM-2DCNN training curve.

The training curve for the ResNet-ICBAM-2DCNN network model is illustrated in Figure 14. In this model, the Adam optimization algorithm and the cross-entropy loss function are utilized for training the two-channel feature fusion. The mean inference time for processing a single signal in this experiment is approximately 12 to 14 milliseconds when using a GPU. The model is configured with a learning rate of 0.0007, a batch size of 256, and a total of 200 training rounds.

### 3.2. Analysis of Results

**Figure 15:**
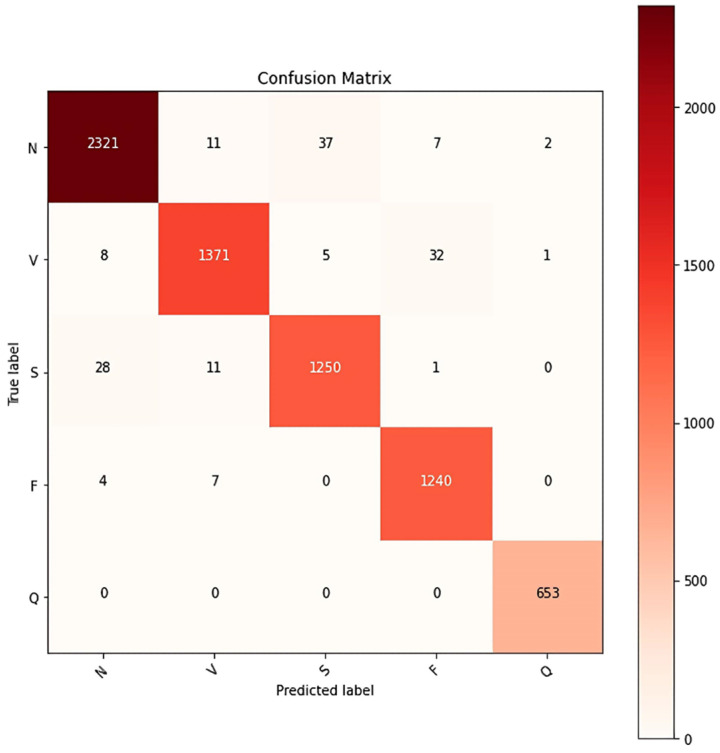
ResNet-ICBAM-2DCNN network model test set classification effect confusion matrix.

After the two-channel feature fusion ResNet-ICBAM-2DCNN network model has been trained and tested using 14,105 sample data units, in which each data unit is not involved in the data augmentation or the training process. The diagonal red squares represent the number of correct classifications, with darker squares representing a larger number of samples. Figure 15 presents the confusion matrix of the ResNet-ICBAM-2DCNN network model for cardiac arrhythmia classification.

The confusion matrix can be used to derive the precision (PPV), sensitivity (Sen), specificity (Spec), F1 value, accuracy (Acc), and overall accuracy (OA) of its model for each category in addition to the sample size, the detailed values of which are shown in Table 5.

The analysis indicated that the network model exhibited varying performance depending on the type of electrocardiogram (ECG) signal data. For class Q ECG signals, all performance metrics were at their highest levels: positive predictive value (PPV) was 0.9954, sensitivity (Sen) was 1.000, F1 score was 0.9977, accuracy (Acc) was 1.0000, and specificity (Spec) was 0.9995. In contrast, identifying class S ECG signals proved more challenging, resulting in slightly lower metrics. This may be attributed to the high similarity of class S signals to other types. When comparing the single-channel ResNet-ICBAM model to the two-channel feature-fused ResNet-ICBAM-2DCNN model, the latter demonstrated improvements across all metrics. Overall, the model achieved a prediction accuracy of 0.9780, reflecting strong performance and good generalization ability in ECG signal prediction.

To verify the effectiveness of the improved convolutional block attention module (ICBAM) and feature fusion, this paper compares the two-channel feature-fused ResNet-ICBAM-2DCNN model with several models to compare the classification results. Among them, ResNet, ResNet-CBAM, and ResNet-ICBAM use one-dimensional convolutional neural networks, while ResNet-2DCNN and ResNet-CBAM-2DCNN combine one- and two-dimensional convolutional neural networks. The classification evaluation metrics of the relevant deep learning models are shown in Table 6.

As shown in Table 4, for the ResNet-ICBAM-2DCNN network model proposed in this paper, compared with other deep learning models, although some metrics are slightly decreased for the ECG signal data samples of type Q, other metrics are significantly improved, and the overall performance is also significantly improved. These clearly show that the performance of the network is improved by adding a 2D convolutional network. The ResNet-ICBAM-2DCNN network model proposed in this paper has excellent performance and can accomplish the classification task effectively.

## 4. Conclusions

For ECG abnormality classification, this paper proposes a method to improve the convolutional block attention module CBAM attention mechanism and combines it with ResNet’s residual connections to build a ResNet-ICBAM network model for recognition and improving its robustness. The process and strategy of building the network model are described. To improve the problem of the classification detection accuracy of its network model for ECG signal data slices, a two-channel feature fusion ResNet-ICBAM-2DCNN network model is also constructed, which performs classification by combining a one-dimensional ResNet-ICBAM network model with a two-dimensional convolutional neural network. The method of visualization of one-dimensional ECG signal slice data, which is specified by Gram angular difference field, Markov variation field, and recurrence map, and the construction method of two-channel feature fusion ResNet-ICBAM-2DCNN network model, as well as the training process, are introduced and tested. After testing, various evaluation indexes are calculated from the test results, and the experimental results show that the dual-channel feature fusion ResNet-ICBAM-2DCNN network model achieves excellent results. Subsequent research will explore multimodal data fusion, enhance model interpretability, and develop real-time monitoring and early warning systems. These efforts will broaden the system’s application and clinical utility. Advancements in these areas will enhance arrhythmia detection and open new avenues for intelligent medicine.

## Figures and Tables

**Table 1:** Before-and-after comparison of sample size augmented by the SMOTE method data.

Heartbeat Type	Pre-Enhancement Sample Size	Enhanced Sample Size
N	9403	9403
V	5935	9403
S	5036	9403
F	4927	9403
Q	2655	9403

**Table 2:** ResNet network architecture.

Stage	Output Size	ResNet-18	ResNet-34	ResNet-50	ResNet-101
Conv1	56 × 56	Cov7 × 7, 64, stride = 2, 3 × 3 Max Plooing			
Conv2_x	56 × 56	$\left[\begin{array}{l} 3\times 3,\;64\\ 3\times 3,\;64 \end{array}\right]\times 2$	$\left[\begin{array}{l} 3\times 3,\;64\\ 3\times 3,\;64 \end{array}\right]\times 3$	$\left[\begin{array}{l} 1\times 1,\;64\\ 3\times 3,\;64\\ 1\times 1,\;256 \end{array}\right]\times 3$	$\left[\begin{array}{l} 1\times 1,\;64\\ 3\times 3,\;64\\ 1\times 1,\;256 \end{array}\right]\times 3$
Conv3_x	28 × 28	$\left[\begin{array}{l} 3\times 3,\;128\\ 3\times 3,\;128 \end{array}\right]\times 2$	$\left[\begin{array}{l} 3\times 3,\;128\\ 3\times 3,\;128 \end{array}\right]\times 4$	$\left[\begin{array}{l} 1\times 1,\;128\\ 3\times 3,\;128\\ 1\times 1,\;512 \end{array}\right]\times 4$	$\left[\begin{array}{l} 1\times 1,\;64\\ 3\times 3,\;64\\ 1\times 1,\;256 \end{array}\right]\times 4$
Conv4_x	14 × 14	$\left[\begin{array}{l} 3\times 3,\;256\\ 3\times 3,\;256 \end{array}\right]\times 2$	$\left[\begin{array}{l} 3\times 3,\;256\\ 3\times 3,\;256 \end{array}\right]\times 6$	$\left[\begin{array}{l} 1\times 1,\;256\\ 3\times 3,\;256\\ 1\times 1,\;1024 \end{array}\right]\times 6$	$\left[\begin{array}{l} 1\times 1,\;256\\ 3\times 3,\;256\\ 1\times 1,\;1024 \end{array}\right]\times 23$
Conv5_x	7 × 7	$\left[\begin{array}{l} 3\times 3,\;512\\ 3\times 3,\;512 \end{array}\right]\times 2$	$\left[\begin{array}{l} 3\times 3,\;512\\ 3\times 3,\;512 \end{array}\right]\times 3$	$\left[\begin{array}{l} 1\times 1,\;512\\ 3\times 3,\;512\\ 1\times 1,\;2048 \end{array}\right]\times 3$	$\left[\begin{array}{l} 1\times 1,\;512\\ 3\times 3,\;512\\ 1\times 1,\;2048 \end{array}\right]\times 3$
	1 × 1	Global Average Pooling, FC			

**Table 3:** The parametric design of the network layer of the ResNet-ICBAM-2DCNN network model.

Network Layer	Input Size	Num Kernels	Kernel Size/Pooling Size	Step Size	Output Size
Input1	(1, 1800)	-	-	-	(1, 1800)
Conv1d Module	(1, 1800)	64	7	2	(64, 900)
Batch Normalization Module	(64, 900)	-	-	-	(64, 900)
MaxPooling1d	(64, 900)	-	3	2	(64, 450)
ICBAM Residuals Module1	(64, 450)	-	-	-	(32, 450)
ICBAM Residuals Module2	(32, 450)	-	-	-	(64, 225)
ICBAM Residuals Module3	(64, 225)	-	-	-	(128, 113)
ICBAM Residuals Module4	(128, 113)	-	-	-	(256, 57)
AdaptiveAvgPool1d	(256, 57)	-	-	-	(256, 1)
Flatten1	(256, 1)	-	-	-	256
Input2	(100, 100, 3)	-	-	-	(100, 100, 3)
Conv2d Module1	(100, 100, 3)	64	7 × 7	1	(94, 94, 64)
MaxPooling2d1	(94, 94, 64)	-	3 × 3	3	(31, 31, 64)
Conv2d Module2	(31, 31, 64)	32	5 × 5	1	(27, 27, 32)
MaxPooling2d2	(27, 27, 32)	-	3 × 3	3	(9, 9, 32)
Conv2d Module3	(9, 9, 32)	16	3 × 3	1	(7, 7, 16)
AdaptiveMaxPool2d	(7, 7, 16)	-	-	-	(3, 3, 16)
Flatten2	(3, 3, 16)	-	-	-	144
Concatenate	400	-	-	-	400
Droupout	400	-	-	-	400
Dense(ReLU)	400	-	-	-	200
Softmax	200	-	-	-	5

**Table 4:** The general form of the confusion matrix.

		Predicted Condition	
		Positive (PP)	Negative (PN)
**Actual Condition**	**Positive (P)**	TP	FN
	**Negative (N)**	FP	TP

**Table 5:** Evaluation index of ResNet-ICBAM-2DCNN network model.

Types of ECG Signals	PPV	Sen	F1	Acc	Spec	Sample Size
N	0.9831	0.9760	0.9795	0.9760	0.9912	2378
V	0.9793	0.9675	0.9734	0.9675	0.9947	1417
S	0.9675	0.9690	0.9682	0.9690	0.9925	1290
F	0.9688	0.9912	0.9798	0.9912	0.9929	1251
Q	0.9954	1.0000	0.9977	1.0000	0.9995	653
OA	0.9780					

**Table 6:** Classification evaluation indicators of related deep learning classification models.

Network Model	Signal Type	PPV	Sen	F1	Acc	Spec	OA
ResNet	N	0.9391	0.8566	0.8960	0.8566	0.9698	0.8990
	V	0.9338	0.8666	0.8990	0.8666	0.9831	
	S	0.7470	0.9248	0.8265	0.9248	0.9264	
	F	0.9452	0.9369	0.9410	0.9369	0.9869	
	Q	0.9775	1.0000	0.9886	1.0000	0.9973	
ResNet-CBAM	N	0.9716	0.8503	0.9069	0.8503	0.9867	0.9162
	V	0.9479	0.9118	0.9295	0.9118	0.9863	
	S	0.7524	0.9612	0.8441	0.9612	0.9268	
	F	0.9653	0.9568	0.9611	0.9568	0.9918	
	Q	0.9924	0.9985	0.9954	0.9985	0.9991	
ResNet -2DCNN	N	0.9675	0.9008	0.9329	0.9008	0.9839	0.9353
	V	0.9677	0.9104	0.9382	0.9104	0.9919	
	S	0.8211	0.9574	0.8840	0.9574	0.9517	
	F	0.9508	0.9736	0.9621	0.9736	0.9883	
	Q	0.9924	0.9985	0.9954	0.9985	0.9991	
ResNet-CBAM-2DCNN	N	0.9885	0.9058	0.9454	0.9058	0.9945	0.9512
	V	0.9697	0.9478	0.9586	0.9478	0.9921	
	S	0.8568	0.9783	0.9135	0.9783	0.9623	
	F	0.9552	0.9880	0.9713	0.9880	0.9894	
	Q	0.9924	1.0000	0.9962	0.9880	0.9992	

## Data Availability

The original contributions presented in this study are included in the article. Further inquiries can be directed to the corresponding author.
